# Ablation Reboots the Response in Advanced Hepatocellular Carcinoma With Stable or Atypical Response During PD-1 Therapy: A Proof-of-Concept Study

**DOI:** 10.3389/fonc.2020.580241

**Published:** 2020-10-09

**Authors:** Ning Lyu, Yanan Kong, Xiaoxian Li, Luwen Mu, Haijing Deng, Huiming Chen, Meng He, Jinfa Lai, Jibin Li, Hailin Tang, Youen Lin, Ming Zhao

**Affiliations:** ^1^Liver Cancer Study and Service Group, Department of Minimally Invasive Interventional Radiology, Sun Yat-sen University Cancer Center, Guangzhou, China; ^2^State Key Laboratory of Oncology in South China, Department of Breast Oncology, Sun Yat-sen University Cancer Center, Guangzhou, China; ^3^Zhongshan School of Medicine, Sun Yat-sen University, Guangzhou, China; ^4^Department of Vascular Interventional Radiology, Third Affiliated Hospital of Sun Yat-sen University, Guangzhou, China; ^5^Department of Clinical Research, Sun Yat-sen University Cancer Center, Guangzhou, China; ^6^State Key Laboratory of Oncology in South China, Sun Yat-sen University Cancer Center, Guangzhou, China; ^7^Department of Interventional Radiology, Jieyang Affiliated Hospital, Sun Yat-sen University, Jieyang, China

**Keywords:** hepatocellular carcinoma, anti-PD-1 mAbs, thermal ablation, nivolumab, pembrolizumab

## Abstract

**Background:** The anti-programmed cell death protein-1 (PD-1) inhibitor is one of the second-line therapies for advanced hepatocellular carcinoma (HCC) after sorafenib failure. The goal of this study is to evaluate the feasibility and safety of ablation on the tumor in patients with advanced HCC who had stable disease or atypical response during single anti-PD-1 therapy after sorafenib failure. Atypical response defined as mixed responses in different lesions of the same individual (e.g., active or stable lesions mixed with progressive lesions).

**Patients and Methods:** This proof-of-concept clinical trial enrolled 50 patients treated with an anti-PD-1 inhibitor of nivolumab or pembrolizumab monotherapy between July 2015 and Nov 2017. Thirty-three cases with stable disease or atypical response to anti-PD-1 inhibitor received subtotal thermal ablation. The safety and the response of ablation during anti-PD-1 therapy were evaluated. The survival was estimated by the Kaplan-Meier curve.

**Results:** Of all 50 patients treated with anti-PD-1 therapy, the rate of response, stable disease, atypical and typical progression were 10% (*n* = 5), 42% (*n* = 21) 32% (*n* = 16), and 12% (*n* = 6), respectively. Additional ablation improved efficacy with tolerable toxicity, and the response rate was increased from 10 to 24% (12/50). The median time to progression, progression-free survival, and overall survival was 6.1 months (95%CI, 2.6–11.2), 5 months (95%CI, 2.9–7.1), and 16.9 months (95%CI, 7.7–26.1), respectively.

**Conclusions:** This proof-of-concept trial suggested that additional ablation may increase the objective response rate with tolerated toxicity and achieved a relatively better median survival, in advanced HCC patients who had stable or atypical progressive diseases during anti-PD-1 therapy, which may provide a potentially promising strategy to treat advanced HCC.

**Trial registration number:** ClinicalTrials.gov identifier: NCT03939975.

## Introduction

Hepatocellular carcinoma (HCC) in advanced stage (Barcelona Clinic Liver Cancer stage-C) is the most frequently diagnosed status, with limited treatment options and high mortality rate (1). Current available treatment for advanced HCC, including atezolizumab plus bevacizumab regimen, multikinase inhibitors (sorafenib, lenvatinib, cabozantinib, and regorafenib), human monoclonal antibodies (ramucirumab), and immune checkpoint inhibitors (nivolumab, pembrolizumab„ and nivolumab plus ipilimumab) have been proven to improve the survivals of patients with advanced HCC by a series of clinical trials (2–7). However, due to the molecular heterogeneity and limited response, the benefits are modest with an extend survival of only a few weeks in second-line treatments, and the progression is still commonly seen.

In recent years, great progress has been made in the field of cancer immunotherapy and encouraging clinical results on many malignancies such as Hodgkin's disease, melanoma, and non-small cell lung cancer and so on raising hopes again for the treatments of advanced HCC (8). Two programmed cell death protein-1 (PD-1) immune checkpoint inhibitors, nivolumab and pembrolizumab, have been approved in second-line setting following sorafenib failure (9, 10). However, not as expected, clinical trials showed that only a small subset, ~17–20% of participants with advanced HCC could respond to monotherapy of anti-PD-1 inhibitor (9, 10). This might be associated with the highly immunosuppressive tumor milieu in advanced HCC (11–13). Researches revealed that a multiplicity of membrane-linked inhibitory molecules [PD-1, cytotoxic T-lymphocyte-associated protein [CTLA]-4, thymocyte selection-associated high mobility group box protein [TOX]] and soluble factors (indoleamine 2,3-dioxygenase, arginase-1, adenosine, and others) involved in the suppression, leading to the exhaustion of antitumor response by T-lymphocytes, finally (8, 14).

Locoregional therapies that are commonly used in HCC have been demonstrated the advantage of boosting the tumor-specific T-cell response by exposing neo-tumor-associated antigens via necrosis of the HCC cells (15–22). We hypothesized that loco-therapies might enhance the response to anti-PD-1 monotherapy, especially in non-sensitive tumors (23, 24). In this proof-of-concept clinical trial, patients with advanced HCC who received single anti-PD-1 inhibitor after sorafenib failure and had a response of stable disease or atypical progression (defined as mixed responses in different lesions of the same individual) were enrolled. We mainly focused on whether the application of subtotal thermal ablation could improve the antitumor response of anti-PD-1 monotherapy.

## Methods

### Participants

This proof-of-concept clinical trial was performed at three hospitals in China with approval of the ethical committee of each participating institution, and all participants provided informed consent. Eligible patients had a pathological diagnosis of HCC by either surgical resection tissue or core needle biopsy and had an advanced stage of a disease that previously received sorafenib or with unacceptable toxicity of sorafenib. Patients with previous organ transplantation, immunodeficient disease, or those who were given immunosuppressive therapies were excluded. Other eligibility criteria included: Child-Pugh A or B7 classification; Eastern Cooperative Oncology Group performance status score 0–2; adequate bone marrow (leukocyte count >3.0 × 10^9^/L, hemoglobin >8.0 g/L, and platelet count >60 × 10^9^/L), liver (alanine aminotransferase and aspartate aminotransferase <200 IU/mL), renal (creatinine <1.5 times the upper limit of the normal range), and coagulation (international normalized ratio <2.3) function.

### Anti-PD-1 Therapy and Ablation Combination Procedures

Nivolumab or pembrolizumab intravenously would be administrated for up to 3 years or until at least 12 months of disease control, intolerable toxicity, or typical disease progression. Nivolumab was given a dose of 3 mg/kg every 2 weeks. Pembrolizumab was given a dose of 3 mg/kg every 3 weeks.

The radiological response was evaluated every 6–8 weeks, as identified by the immune-related Response Evaluation Criteria in Solid Tumors (RECIST) (25). In brief, the cutoff values of complete response (disappearance of all lesions), partial response (≥30% decrease of the sum of the longest diameters of target lesions from baseline) and progressive disease (≥20% increase from baseline) by RECIST were used. Progressive diseases were divided into two categories: typical progression and atypical progression. Atypical progression was the context of distinct responses occurring in different lesions in the same patient (e.g., active or stable lesions mixed with progressive lesions). Patients with stable diseases or atypical progression to anti-PD-1 monotherapy would be additionally treated with subtotal thermal ablation along with immunotherapy; and for those who with no lesions eligible for ablation, immunotherapy would be given solely. Patients with complete or partial responses would also keep on going with immunotherapy. Others with typical progression would stop immunotherapy.

Subtotal radiofrequency ablation (RFA) or microwave ablation (MWA) would be performed with computed tomography guidance within 10–14 days of radiological assessment and be followed by immunotherapy within 3–7 days. The anti-PD-1 inhibitor should be as same as those performed before ablation. Subtotal ablation defined as that up to two lesions (either intrahepatic or extrahepatic) was adequately ablated in one treated procedure, leaving most of the other lesions untreated. The lesion chosen for ablation was treated with curative intent and selected with consideration of minimizing technical risks, such as avoiding damage of large vessels, gastrointestinal tracts, among other structures. For patients assessed with atypical progression after 3 months of ablation, repeated subtotal ablation was allowed. Details of computed tomography-guided RFA or MWA were described in the Supplementary Methods (26, 27).

### Safety and Efficacy

Safety evaluation was done continuously during immunotherapy and up to 90 days after the last dose by using the Common Terminology Criteria for Adverse Events (version 4.0). Complications related to ablation procedure were assessed during the next (0–24 h) and periprocedural (1–30 days) period and reported according to the Society of Interventional Radiology Classification System for Complications (28). Efficacy included an objective response (includes complete and partial response), duration of response, and disease control (Includes complete and partial response, and stable disease for at least 3 months).

### Outcomes

The primary objective was the feasibility of systemic anti-PD-1 therapy in combination with loco-ablation in patients with advanced HCC for which anti-PD-1 monotherapy could not achieve a satisfactory response. The study mainly involved two aspects of feasibility: safety and efficacy. Secondary objectives were the time to tumor progression (TTP; time from the first dose of anti-PD-1 drug until the first typical disease progression), progression-free survival (PFS; time from first day of immunotherapy to first typical disease progression, or death, which occurred earlier) and overall survival (OS; time from first immunotherapy to death of any cause). Patients were followed up for survival every 4–6 months. An exploratory objective was the tumor growth kinetics (TGK) before and during immunotherapy. The method of TGK calculation was recorded in the Supplementary Methods.

### Statistics

A sample size of about 50 subjects was chosen for the study to provide a reasonably reliable estimate of efficacy and sufficient safety or complications follow up. Baseline characteristics and adverse events (AEs) were summarized with descriptive statistics. Safety was assessed in all enrolled patients who received at least one dose of anti-PD-1 inhibitor. Duration of response, TTP, PFS, and OS were estimated by the Kaplan-Meier curve and reported along with 95% confidence interval (CI). Data were analyzed with SPSS version 25.0. All data of this study have been recorded at the study center (number RDDA2017000320). The ClinicalTrials.gov identifier number was NCT03939975.

## Results

### Patient Characteristics

Between July 2015, and Nov 2017, fifty patients were enrolled in the study treated with an anti-PD-1 monotherapy. Two patients had drug discontinuation by serious AEs before the first image examination and were assessed for safety only. Thirty-seven patients had stable or atypical progressive diseases to anti-PD-1 monotherapy; three of the 37 patients had no tumors suitable for ablation, and another one patient declined to undergo ablation treatment; thus, a total of 33 patients were treated with additional ablation.

Patients baseline characteristics in the study were summarized in Table 1. Either macrovascular invasion or extrahepatic metastases were present in 45 (90%) patients. All the patients were heavily pretreated by multiple therapies and had experiences of receiving sorafenib. In terms of the most recent treatment ahead of anti-PD-1 therapy, 28 (56%) of the 50 patients were treated with sorafenib, 12 (24%) with arterial infusion chemotherapy of oxaliplatin and fluorouracil, 6 (12%) with TACE, and 4 (8%) with lenvatinib; 41 (82%) patients had discontinued such therapies due to disease progression, and nine (18%) patients had discontinued due to treatment-related toxicities or technical factors (includes six by sorafenib, two by TACE, and 1 by lenvatinib). The median time interval between recent therapy stopping and anti-PD-1 therapy commencement was 1.9 months (range, 1.1–3.2).

**Table 1:** Baseline characteristics.

	***n* = 50**
Age ^†^ (years)	51 (19–74)
**Gender**	
Male	46 (92)
**Etiology**	
Hepatitis B virus	46 (92)
Hepatitis C virus	0 (0)
Others	4 (8)
**Child-Pugh class/score**	
A	46 (92)
B	4 (8)
**ECOG performance status**	
0	16 (32)
1	34 (68)
A-fetoprotein level ^†^ (ng/ml)	269.5 (0.97–12.1 × 10^4^+)
>400 ng/ml	23 (46)
<400 ng/ml	27 (54)
**No. of Tumor**	
≤ 5	10 (20)
>5, ≤ 10	15 (30)
>10	25 (50)
**Portal invasion**	
Absent	30 (60)
Present	20 (40)
**Extrahepatic metastases**	
Absent	13 (26)
Present	37 (74)
Lung	27 (54)
Lymph node	12 (24)
Bone	5 (10)
Adrenal gland	3 (6)
**Portal invasion or extrahepatic metastases**	
Absent	5 (10)
Present	45 (90)
**Previous treatment**	
Surgical resection	27 (54)
Thermal ablation	22 (44)
TACE	29 (58)
HAIC	17 (34)
Sorafenib	50 (100)
Lenvatinib	5 (10)
Regorafenib	1 (2)
Radiotherapy	3 (6)
**Recent treatment**	
Therapy	
Sorafenib	28 (56)
HAIC	12 (24)
TACE	6 (12)
Lenvatinib	4 (8)
**Reason for discontinuation**	
Disease progression	41 (82)
Toxicity	9 (18)

*Data are n (%), unless otherwise indicated*.

† *Data are expressed medians. Numbers in parentheses are ranges*.

*ECOG, Eastern Cooperative Oncology Group; HAIC, hepatic arterial infusion of chemotherapy; TACE, transarterial chemoembolization*.

**Figure 1:**
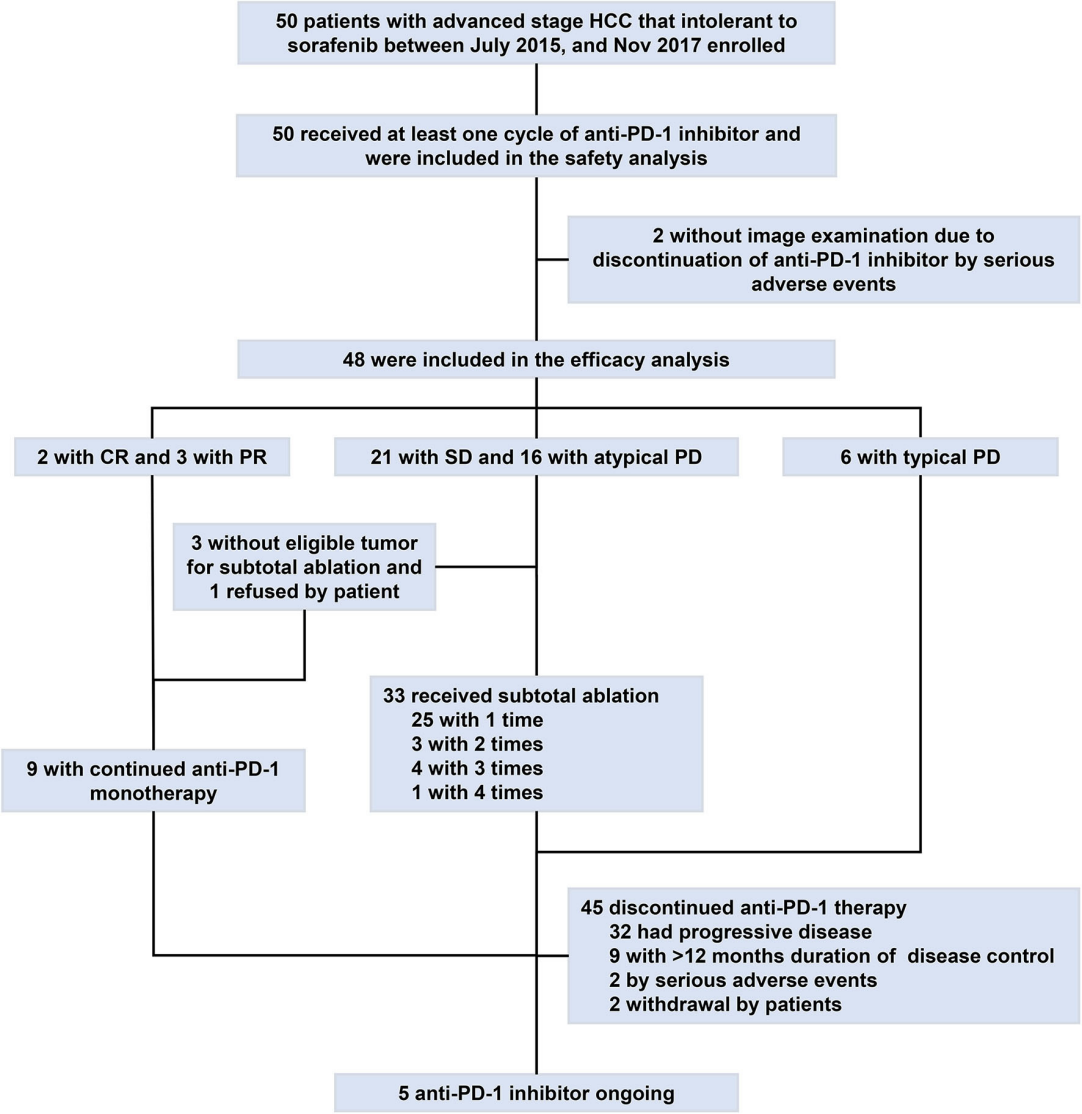
Study profile flow. CR, complete response; HCC, hepatocellular carcinoma; PD-1, programmed cell death protein-1; PD, progressive disease; PR, partial response; SD, stable disease.

Thirty-three (66%) of the 50 patients experienced with ablation, among whom, eight (24.2%) patients experienced two or more times of ablation due to repeated atypical disease progression included 3 (9.1%) experienced two times, 4 (12.1%) experienced three times, and 1 (3%) experienced four times. With a median follow-up of 17.9 months (range, 4.6–41.6) by Mar 31, 2019, 47 (94%) of the 50 patients discontinued immunotherapy. The median duration of immunotherapy was 6.5 months (range, 1.6–32.4). Most patients discontinued immunotherapy due to disease progression (*n* = 32; 64%) or duration of disease control longer than 12 months (*n* = 9; 18%) (Figure 1).

### Safety

Treatment-related AEs for both anti-PD-1 inhibitor and ablation therapy were recorded in Table 2. At least one anti-PD-1 inhibitor-related toxicity has occurred in 41 (82%) of the 50 patients and, among those, 7 (14%) were as serious AEs. AEs of any grade that occurred in at least 10% of patients were fatigue in 17 (34%) patients, transaminitis in 10 (20%) patients, fever in 8 (16%) patients, diarrhea in 6 (12%) patients, and pneumonitis in 5 (10%) patients. The most frequent serious AEs was hyperbilirubinemia in two (4%) patients. No cases of fulminant increases of the hepatitis B virus (HBV) were recorded. Four (8%) of the 50 patients had to discontinue immunotherapy due to AEs. One patient experienced grade four pneumonitis, which occurred after the third dose of pembrolizumab and died after 4 weeks of immunotherapy discontinuation. One patient discontinued pembrolizumab because of grade 4 of hyperbilirubinemia. One patient discontinued nivolumab because of grade 3 of thyroid dysfunction. One patient developed slowly increased creatinine level (max of 2.3 mg/dL) and discontinued pembrolizumab with a total of 14 doses but remained tumor control for 19.7 months by the date cutoff.

**Table 2:** Treatment-related adverse events.

		***n* = 50**	
**Anti-PD-1 inhibitor-related AEs**	**Any grade**	**Grade 1–2**	**Grade 3–4**
Discontinued due to AEs	4 (8)	1 (2)	3 (6)
Fatigue	17 (34)	17 (34)	0
Transaminitis	10 (20)	10 (20)	0
Fever	8 (16)	8 (16)	0
Diarrhea	6 (12)	6 (12)	0
Pneumonitis	5 (10)	4 (8)	1 (2)
Hyperbilirubinemia	4 (8)	2 (4)	2 (4)
Hypothyroidism	4 (8)	3 (6)	1 (2)
Pruritus	4 (8)	4 (8)	0
Rash	4 (8)	4 (8)	0
Hyperthyroidism	3 (6)	3 (6)	0
Hypoalbuminemia	3 (6)	3 (6)	0
Hypoleukemia	3 (6)	2 (4)	1 (2)
Thrombocytopenia	3 (6)	2 (4)	1 (2)
Prolactin increase	2 (4)	2 (4)	0
Alopecia	1 (2)	1 (2)	0
Anemia	1 (2)	1 (2)	0
Appetite decrease	1 (2)	1 (2)	0
Creatinine increase	1 (2)	1 (2)	0
Diabetic metabolic decompensation	1 (2)	1 (2)	0
Nausea	1 (2)	1 (2)	0
		***n*** **=** **47** ^*^	
**Ablation-related complications**	**Any**	**Grade A-B**	**Grade C-D**
Discontinued due to complications	0	0	0
Pain	47 (100)	41 (87.2)	6 (12.8)
Transaminitis	19 (40.4)	10 (21.3)	9 (19.1)
Vomiting	22 (46.8)	22 (46.8)	0
Constipation	13 (27.7)	13 (27.7)	0
Fever	11 (23.4)	11 (23.4)	0
Intraabdominal hemorrhage	11 (23.4)	9 (19.1)	2 (4.3)
Pneumothorax	7 (14.9)	5 (10.6)	2 (4.3)
Pleural effusion	9 (19.1)	7 (14.9)	2 (4.3)
Bile duct pneumatosis	6 (12.8)	6 (12.8)	0

*Data are n (%), unless otherwise indicated*.

* *A total of 47 times of ablation procedures were performed in 33 patients*.

*AEs, adverse events; PD-1, programmed cell death protein-1*.

A total of 47 times of ablation procedure was performed in the 33 patients treated with combined therapy. No ablation-related severe complications (Grade E) or death (Grade F) were recorded within 30 days of the ablation procedure. There was also no immunotherapy interruption directly attributable to the ablation procedure. Most of the ablation-related complications were common in routine clinical practice and managed as per the standard of care (Table 2). Transaminase increase (Grade C) was the most frequent major complication occurred in 9 (19.1%) of the 47 ablation sessions.

### Efficacy

**Figure 2:**
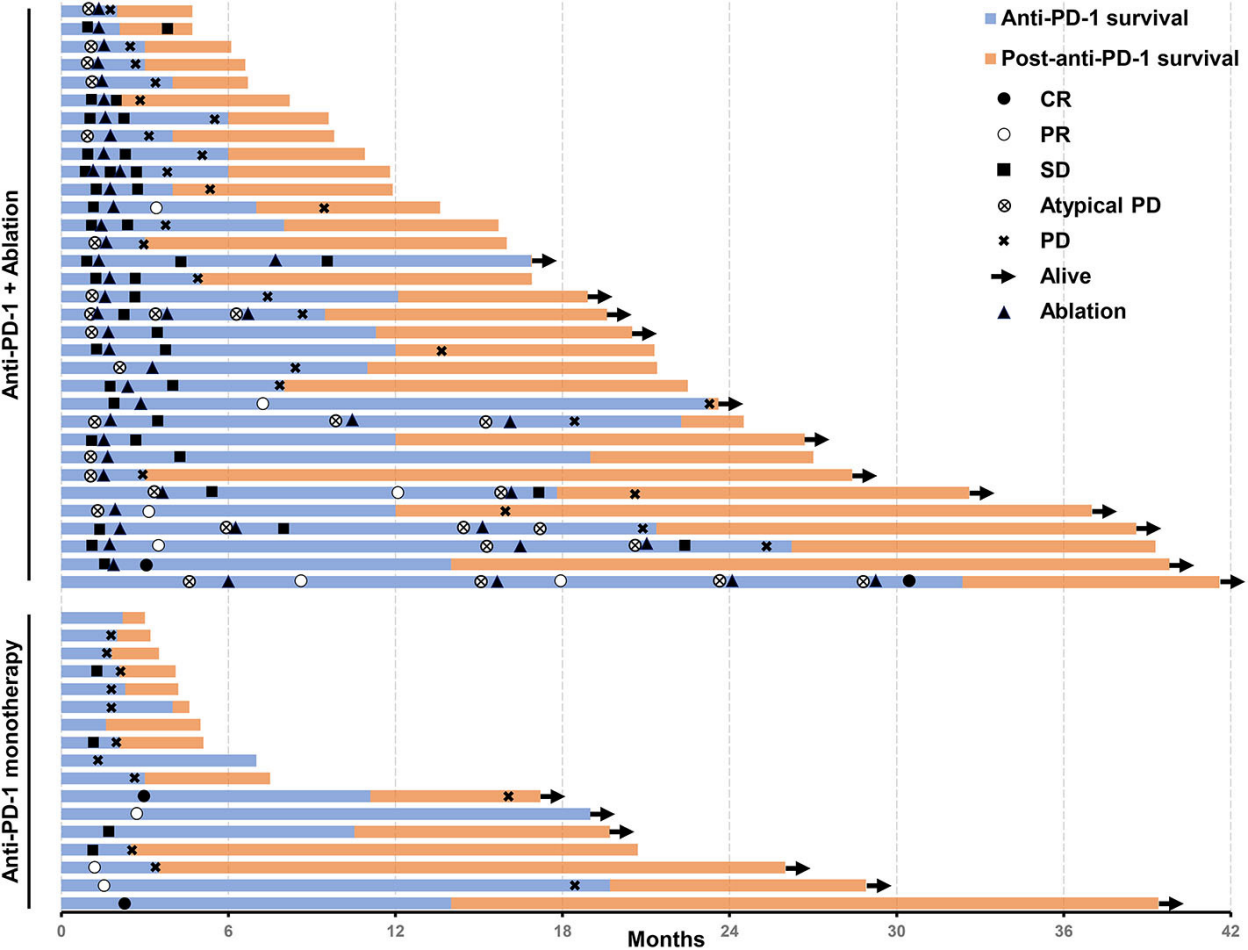
Swimmer's plot shows the time of response, time of ablation, the survival of patients treated with an anti-PD-1 inhibitor in the combination of thermal ablation or anti-PD-1 monotherapy, post-discontinuation of anti-PD-1 treatment survival, and current status. Assessed in a total of 50 patients. PD-1, programmed cell death protein-1.

**Figure 3:**
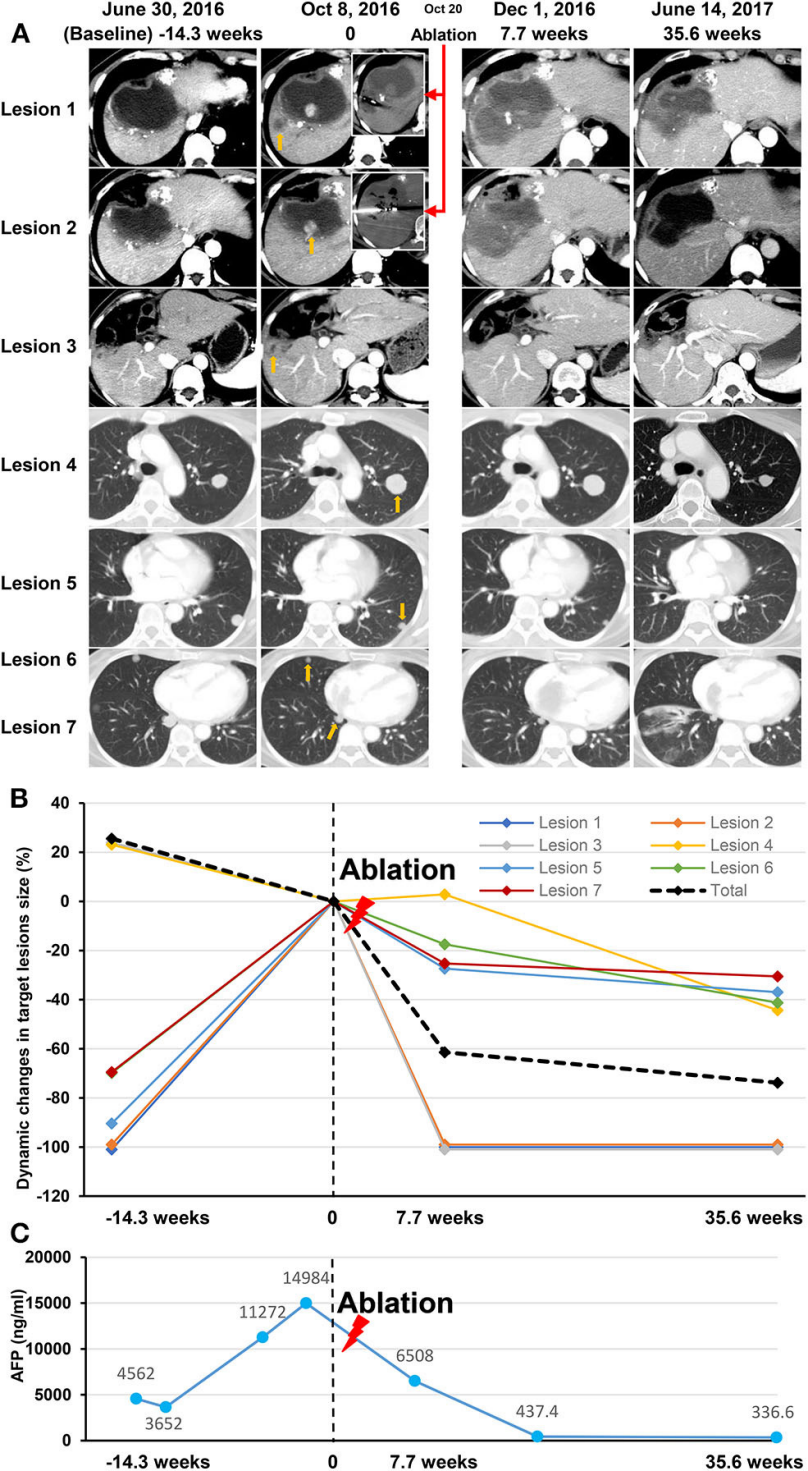
Data of a participant treated with an anti-PD-1 inhibitor in the combination of subtotal thermal ablation. The patient had a diagnosis of recurrent intrahepatic tumors with tumor thrombus invading both inferior vena cava and right atrium, and multiple lung metastases. After receiving 14.3 weeks of anti-PD-1 inhibitor of pembrolizumab, an atypical progression was assessed on image examination on October 8, 2016 (time of 0) as that intrahepatic tumors (Lesion 1, 2, and 3; yellow arrow), vascular invasions (no showing), and part of the lung lesions progressed (a representative example as Lesion 4; yellow arrow), but the other part of lung metastases (representative examples as Lesion 5, 6, and 7; yellow arrow) regressed. Two progressive lesions (Lesion 1 and Lesion 2) in the liver was selected for subtotal thermal ablation (red arrows), and consequently, the leaving intrahepatic tumor (Lesion 3) disappeared, and all the lung metastases regressed due to the combination of pembrolizumab and ablation therapy. Stable disease was recorded on December 1, 2016 (7.7 weeks), and a partial response was achieved on June 14, 2017 (35.6 weeks). Pembrolizumab infusion was lasted for 17.8 months and discontinued because of more than 12 months of ongoing disease control. At last follow-up, the patient was still alive with a progression-free survival of 21.4 months and overall survival of 32.6 months. **(A)** The Images of seven lesions at baseline, response assessment to anti-PD-1 monotherapy, ablation procedure, and post-ablation assessment. **(B)** Dynamic changes in the size of the seven lesions before and after thermal ablation (red lighting). **(C)** The dynamic curve of the serum AFP level before and after thermal ablation (red lighting). AFP, alfa-fetoprotein; PD-1, programmed cell death protein-1.

**Figure 4:**
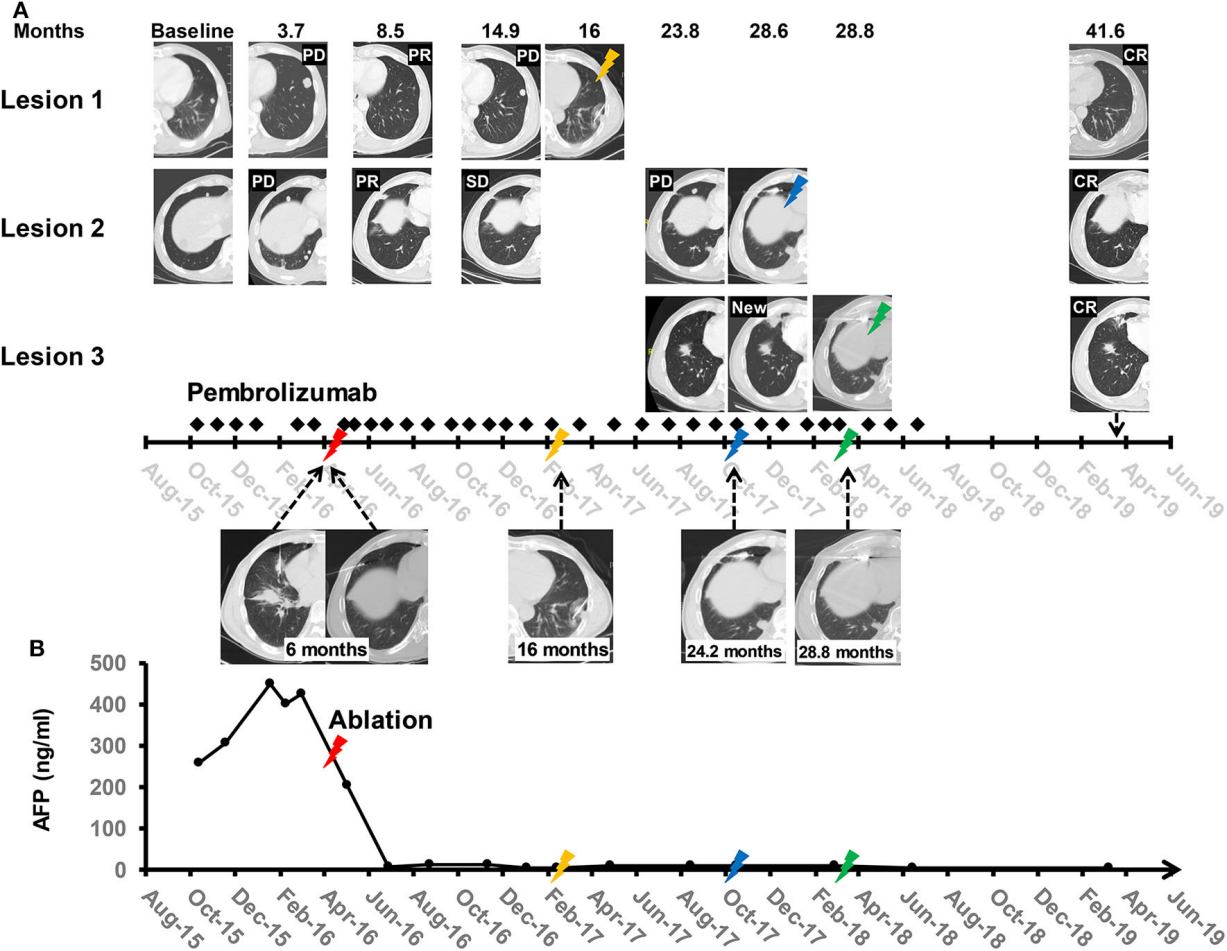
Clinical events of a participant who treated with a continuous anti-PD-1 inhibitor of pembrolizumab in the combination of multiple sessions of thermal ablation. The patient was enrolled due to progressive lung metastases to sorafenib and had an atypical response (stable lesions with progressive lesions) to pembrolizumab monotherapy after 6 months of anti-PD-1 inhibitor initiated. Then the first subtotal ablation (red lighting) was performed, and the size of two targeted lesions (Lesion 1 and 2) shrunk obviously after 2.5 months of ablation. The duration of response of Lesion 1 and Lesion 2 since the first ablation was 14.9 months and 23.8 months, respectively. Lesion 1 (yellow lighting) and Lesion 2 (blue lighting) were ultimately ablated due to tumor progression. The fourth session of ablation (green lighting) was done for a new tumor (Lesion 3), which occurred at 28.6 months from baseline. A total of 33 doses of pembrolizumab was infused with a duration of 32.4 months. At date cutoff, the patient had a complete response to anti-PD-1 inhibitor in the combination of ablation, with a level of serum alfa-fetoprotein in the normal range, and progression-free survival of 41.6 months. **(A)** The middle panel shows the timeline of treatments, including pembrolizumab (black rhombus) and ablation (lightning). Upper panels show CT images of three representative lesions at baseline, course of treatment, and last follow-up since initiation of the pembrolizumab. The lower panel shows CT images of the four sessions of ablation. CR, complete response; PD-1, programmed cell death protein-1; PD, progressive disease; PR, partial response; SD, stable disease. **(B)** The dynamic curve of the serum AFP level. AFP, alfa-fetoprotein.

An objective response was detected in five (10%) of the 50 patients who were treated with anti-PD-1 monotherapy. Twenty-one (42%) patients had stable diseases, 16 (32%) patients had atypical progressive diseases, and 6 (12%) had typical progressive diseases. Two patients (4%) died before the first image examination due to serious AEs. Thirty-seven patients (includes 21 with stable diseases and 16 with atypical progressive diseases) were preliminary candidates for thermal ablation; three of the 37 candidates could not be treated because they did not have eligible tumors for ablation and one candidate declined to receive ablation. Ultimately, ablation was performed in 33 patients, and the technical success rate was 100%. Seven (21.2%) of the 33 patients were recorded improved efficacy by combined therapy included 2 (6.1%) with a complete response and 5 (15.1%) with partial response. Thus, the objective response rate (ORR) of the 50 patients was increased to 24% (12 in 50 patients) by treating with the combined therapy. The best changes from baseline in sizes of the targeted lesions were shown in Supplementary Figure 1. At data cutoff, 5 (41.7%) responders were ongoing, and the median duration of response of the 12 responders was 21.4 months (95%CI, 14.7–28.1). Disease control was detected in 30 (60%) of the 50 patients with combined therapy (Table 3). Figure 2 showed efficacy and survival for the participants on the study in addition to the response to treatment, time of ablation, and duration of immunotherapy. Figure 3 described the images of radiological examinations and subtotal ablation, and target tumor growth kinetics, and alpha-fetoprotein dynamics of a patient who achieved a durable response to combined therapy. Figure 4 summarized the clinical events of a patient who treated with a continuous immunotherapy in the combination of multiple sessions of ablation.

**Table 3:** Response to anti-PD-1 monotherapy or combined therapy.

**Response**	**Anti-PD-1**	**Anti-PD-1 + ablation**
	**(*n* = 50)**	**(*n* = 50)**
**BEST RESPONSE**		
Complete response	2 (4%)	4 (8%)
Partial response	3 (6%)	8 (16%)
Stable disease	21 (42%)	22 (44%)
Progressive disease	22 (44%)	14 (28%)
Not assessable	2 (4%)	2 (4%)
Objective response ^†^	5 (10%)	12 (24%)
Disease control ^‡^	-	30 (60%)
Median DOR, months (95% CI) ^||^	-	21.4 (14.7–28.1)
Median TTP, months (95% CI)	-	6.1 (2.6–11.2)
Median PFS, months (95% CI)	-	5 (2.9–7.1)
Median OS, months (95% CI)	-	16.9 (7.7–26.1)

*Data are n (%), unless otherwise indicated*.

† *Includes complete response and partial response*.

‡ *Includes complete response, partial response and stable disease for at least 3 months*.

|| *Assessed in patients with complete responses or partial responses*.

*CI, confidence interval; DOR, duration of response; OS, overall survival; PD-1, programmed cell death protein-1; PFS, progression-free survival; TTP, time to progression*.

### Outcomes

Forty-one (82%) of the 50 patients had disease progression or died until the last follow-up. The median TTP was 6.1 months (95%CI, 2.6–11.2), and the median PFS was 5 months (95%CI, 2.9–7.1). Thirty-two (64%) patients had died, and the median OS was 16.9 months (95%CI, 7.7–26.1) (Supplementary Figure 2). The estimated 6-, 12-, and 24-months PFS rates of the 50 patients were 44, 34, and 11.9%, respectively. The estimated 6-, 12-, and 24-months OS rates were 78, 56, and 35.9%, respectively. The median PFS [16.4 months [95% CI, 7.1–25.7] vs. 2.6 months [2.2–3.0]; hazard ratio [HR], 0.181 [95% CI, 0.9–0.364]; *P* < 0.001] and median OS [27 months [11.5–42.5] vs. 6.6 months [5.3–7.9]; 0.228 [0.109–0.478]; *P* < 0.001] was significantly longer in patients with disease control (lasted at least 3 months) compared with those who without (Supplementary Figure 3).

In the exploratory analysis, we compared TGK on the last treatment ahead of anti-PD-1 treatment and TGK on anti-PD-1 treatment. Forty-one patients had tumors that were evaluable for TGK calculation both on last treatment and immunotherapy, among them, 4 (9.8%) patients had TGK_R_ ≥ 2, 2 (4.9%) patients had TGK_R_ between 1 and 2, 21 (51.2%) patients had TGK_R_ between 0 and 1, and 14 (34.1%) patients had TGK_R_ <0 (Supplementary Figure 4). At date cutoff, 3 of the four patients with TGK_R_ ≥ 2 had died and had a poor OS of 3.5, 4.7, and 6.7 months, respectively; another one patient switched to receiving lenvatinib and was still alive with a survival of 28.4 months.

## Discussion

This proof-of-concept study investigated the feasibility and safety of the combination of anti-PD-1 inhibitors and thermal ablation in appropriate lesions of patients with advanced HCC after sorafenib failure. We found that in patients who had stable or atypical progressive diseases during immunotherapy, additional ablation could increase the ORR with tolerated toxicity and achieved a relatively better median survival, indicating that ablation may stimulate and enhance the antitumor immunity of anti-PD-1 therapy.

Nivolumab and pembrolizumab were acceleratedly approved to be used in the second line treatment for advanced HCC after sorafenib failure in recent 2 years (29). CheckMate-040 proved that the ORR of Nivolumab was 15–20% in advanced HCC (9) and Keynote-240 reported an ORR of 18.3% for pembrolizumab (30). In our study, we observed an ORR of only 10% in patients who received nivolumab or pembrolizumab, which might be attributed to two reasons, firstly, CheckMate 040 and Keynote-240 were purely second line studies and post sorafenib while in our study all patients were exposed to sorafenib but sorafenib was not the only proceeding therapy prior to anti-PD-1 antibody; Secondly, the etiology of population-92% patients in this study had HBV infection, while the percentage in Checkmate-040 and Keynote-240 were 23.8 and 25.9%, respectively. The subgroup analysis of Checkmate-040 showed that the ORR in HBV-infection population was 7%, which was near to ours.

Ablation is one approach of loco-therapies and commonly used for HCC (31, 32). In recent years there has been an increasing wariness that loco-therapies may eliminate not only tumors but also have additional systemic effects (16, 33). Some studies described the immunological “abscopal effects” induced by loco-therapies and a range of cytokine and chemokine changed following various ablative procedures, suggesting that once the immune response is triggered the effects could be potentially amplified by immunotherapy (16, 17, 34, 35). Our study confirmed the hypothesis and found additional ablation enhanced the antitumor effects of anti-PD-1 inhibitors and increased the response rate. Repeated ablations were also proved feasible and safe. Moreover, the efficacy was not limited in the lesion which treated with ablation but observed in the outside zone, indicating that the systemic effects brought by ablation indeed exist. Shi L et al. demonstrated that in liver metastases from colorectal cancer, tumor quickly overcame T-cell-mediated immune responses which were triggered by RFA of one tumor initially by inhibiting the function of CD8^+^ and CD4^+^ T-cells, driving a shift to higher regulatory T-cell to effector T-cell ratio, and upregulating PD-L1/PD-1 expression (19). For MWA, broad analysis of circulating cytokines proved that the production of IL-12, a Th1 cytokine, is enhanced after MWA however the secretion of Th2 cytokines IL-4 and IL-10 is inhibited, leading to a positive antitumor response. (36) PD-L1-PD-1 axis might play a critical role in ablation-induced antitumor immune responses, which need to be further validated in advanced HCC (24, 37).

A recent study conducted by Greten et al. firstly reported that a combination of tremelimumab, an anti-CTLA-4 inhibitor with ablation in heavily pretreated post-sorafenib population was feasible and resulted in objective tumor responses outside of the ablated zone (38). However, all the patients in that study were treated with ablation, unselectively, leading to a significant question that whether the ablation or tremelimumab itself or both account for the antitumor effects. Our study may give some reference to this question. Although all 50 patients received immunotherapy, those who would be treated with ablation depend on the response to immunotherapy. Eleven patients were excluded, including 5 (10%) patients with objective response to anti-PD-1 monotherapy and 6 (12%) with typical progressive diseases, in which situation we regarded ablation not necessary. Finally, 33 patients with stable or atypical progressive diseases during anti-PD-1 monotherapy underwent ablation. This selectivity of the population is significant to judge the value of loco-therapies during immunotherapy (39, 40). Our study found that 7 (21.2%) of the 33 patients were recorded improved efficacy including 2 (6.1%) with a complete response and 5 (15.1%) with partial response. The ORR of all 50 patients was increased from 10 to 24% after treated with the combined therapy, indicating that ablation combined with the immunotherapy is feasible in patients who had stable or atypical progressive diseases during anti-PD-1 monotherapy.

There are still some limitations in our study. Firstly, the sample size is not large enough that may lead to bias; Secondly, the population enrolled in this study was mainly with HBV-infection, accounting for 92% of all patients, leading to the results valuable in part of patients with advanced HCC; Thirdly, in our study, all patients were exposed to sorafenib but not all received anti-PD-1 antibody immediately post sorafenib failure and 44% of patients received systemic therapies more than sorafenib, which we think it should be noticed.

In conclusion, this proof-of-concept trial suggested that additional thermal ablation combined with anti-PD-1 inhibitors increased the response rate and improved survival in patients with advanced HCC after sorafenib failure who had a stable or atypical progressive disease during anti-PD-1 monotherapy, which may provide a potentially promising strategy to treat advanced HCC.

## Data Availability Statement

The raw data supporting the conclusions of this article will be made available by the authors, without undue reservation.

## Ethics Statement

The studies involving human participants were reviewed and approved by The Ethical Committees of Sun Yat-Sen University Cancer Center, Third Affiliated Hospital of Sun Yat-Sen University, and Jieyang Affiliated Hospital, Sun Yat-Sen University. The patients/participants provided their written informed consent to participate in this study.

## Author Contributions

MZ, NL, YK, LM, and JLi conceived and designed the study. NL, LM, XL, HC, YL, JLa, MH, and HD collected the data. NL, YK, LM, XL, JLi, HT, and MZ analyzed and interpreted the data. All authors were involved in the drafting, review, and approval of the report and the decision to submit for publication.

## Conflict of Interest

The authors declare that the research was conducted in the absence of any commercial or financial relationships that could be construed as a potential conflict of interest.

## Abbreviations

AEs: adverse events
CI: confidence interval
CTLA: cytotoxic T-lymphocyte-associated protein
HBV: hepatitis B virus
HCC: hepatocellular carcinoma
HR: hazard ratio
MWA: microwave ablation
OS overall survival
ORR: objective response rate
PD-1: programmed cell death protein-1
PFS: progression-free survival
RECIST: Response Evaluation Criteria in Solid Tumors
RFA: radiofrequency ablation
TGK: tumor growth kinetics
TOX: thymocyte selection-associated high mobility group box protein
TTP: time to tumor progression.
