# Supplementary material for: Ablation Reboots the Response in Advanced Hepatocellular Carcinoma With Stable or Atypical Response During PD-1 Therapy: A Proof-of-Concept Study
Source: Front Oncol. 2020 Oct 9;10:580241. doi: 10.3389/fonc.2020.580241 (PMC7581675; doi:10.3389/fonc.2020.580241)
Supplement: Supplementary file 1 [file Data_Sheet_1.docx]

Supplement to: **Ablation reboots the response in advanced hepatocellular carcinoma with stable or atypical response during PD-1 therapy: A proof-of-concept study**

# Supplementary methods

Computed tomography-guided ablation technique

All tumor ablations were performed percutaneously under computed tomography guidance by one interventional radiologist (xxx with approximately 15 years of experience in oncologic tumor thermal ablation). Detailed information regarding tumor ablation, including the potential influence of tumor ablation to immunotherapy, treatment process, and procedurally related complications, was carefully explained to patients before enrollment.

The ablative session was described in our previous studies [26, 27, 39]. In brief, the entry site of the skin and the trajectory of applicator were designed in consideration of avoiding injury to nearby vital structures, such as the gastrointestinal tract, large vessels, and adjacent organs. After administration of local anesthesia and intravenous conscious sedatives, a mono-applicator was stepwise inserted into the lesion and positioned against lesion’s most in-depth margin. The ablation session with radiofrequency ablation or microwave ablation devices was performed according to the manufacturer’s instructions.

For radiofrequency ablation, monopolar electrode (STARmed, Goyang-si, Gyeonggi-do, Korea) with a 2-cm or 3-cm active tip was applied mainly based on tumor size. The ablative energy was set at an estimated average power of 150 watts (range, 100 to 200 watts). Grounding pads were commonly adhered to the smooth skin of the patient according to the manufacturer’s specifications.

For microwave ablation, an antenna (Weijing Microwave Electronic Institute, Nanjing, China) with an internally cooled applicator was used. The ablative power was set at an average of 60 watts (range, 50 to 70 watts). The temperature of the antenna was maintained at 10℃ ± 2 (standard deviation) by using a peristaltic pump (BT01-100 LanGe-Pump; LanGe Steady Flow Pump, Baoding, China) in which chilled saline solution recirculate at a rate of 50 to 60 ml/min.

In general, the mean duration of per site application was about 12 minutes (range, 10 to 15 minutes) for both ablation techniques. The treated procedure was finished when the ablation zone achieved an overlapping margin beyond the long axis of the tumor. All trajectories of applicator would be cauterized to prevent potential bleeding or tumor seeding. After ablation, the diagnostic scan was performed immediately to monitor treatment-related complications and technical success. If the residual tumor existed, repeated ablations were performed during the same session.

Technical success was defined that the lesion referred to ablation was treated according to the protocol, and the ablation zone adequately covered tumor plus a safe margin during the procedure. Technique efficacy was defined as all target lesions subjected to ablation achieving complete responses as evidenced by image examination 3-4 weeks after ablation session.

Calculation of tumor growth kinetics (TGK)

To exploratory compare, TGK before and during anti-programmed cell death protein-1 (PD-1) inhibitors or that before and after subtotal ablation, the sum of the largest diameters of target lesions according to Response Evaluation Criteria in Solid Tumors criteria over time was calculated using the algorithm described in a previous study [40]. In brief, *T_Before_*, *T_0_*, and *T_After_* represent the time of tumor assessment before-baseline, baseline, and first imaging after baseline, respectively. *S_Before_*, *S_0_*, and *S_After_* represent the sum of the largest diameter of target lesions before-baseline, baseline, and first imaging after baseline, respectively. TGK_Before_ was defined as (*S_0－_S_Before_*)/(*T_0－_T_Before_*). TGK_After_ was defined as (*S_After－_S_0_*)/(*T_After－_T_0_*). TGK ratio (TGK_R_) was defined as TGK_After_/ TGK_Before_. Lesions subjected to subtotal ablation were not included in the sum of measurement.

References:

1. Pan T, Xie QK, Lv N et al. Percutaneous CT-guided Radiofrequency Ablation for Lymph Node Oligometastases from Hepatocellular Carcinoma: A Propensity Score-matching Analysis. Radiology 2017; 282: 259-270.

2. Mu L, Pan T, Lyu N et al. CT-guided percutaneous radiofrequency ablation for lung neoplasms adjacent to the pericardium. Lung Cancer 2018; 122: 25-31.

3. Mu L, Sun L, Pan T et al. Percutaneous CT-guided radiofrequency ablation for patients with extrahepatic oligometastases of hepatocellular carcinoma: long-term results. Int J Hyperthermia 2018; 34: 59-67.

4. Saada-Bouzid E, Defaucheux C, Karabajakian A et al. Hyperprogression during anti-PD-1/PD-L1 therapy in patients with recurrent and/or metastatic head and neck squamous cell carcinoma. Ann Oncol 2017; 28: 1605-1611.


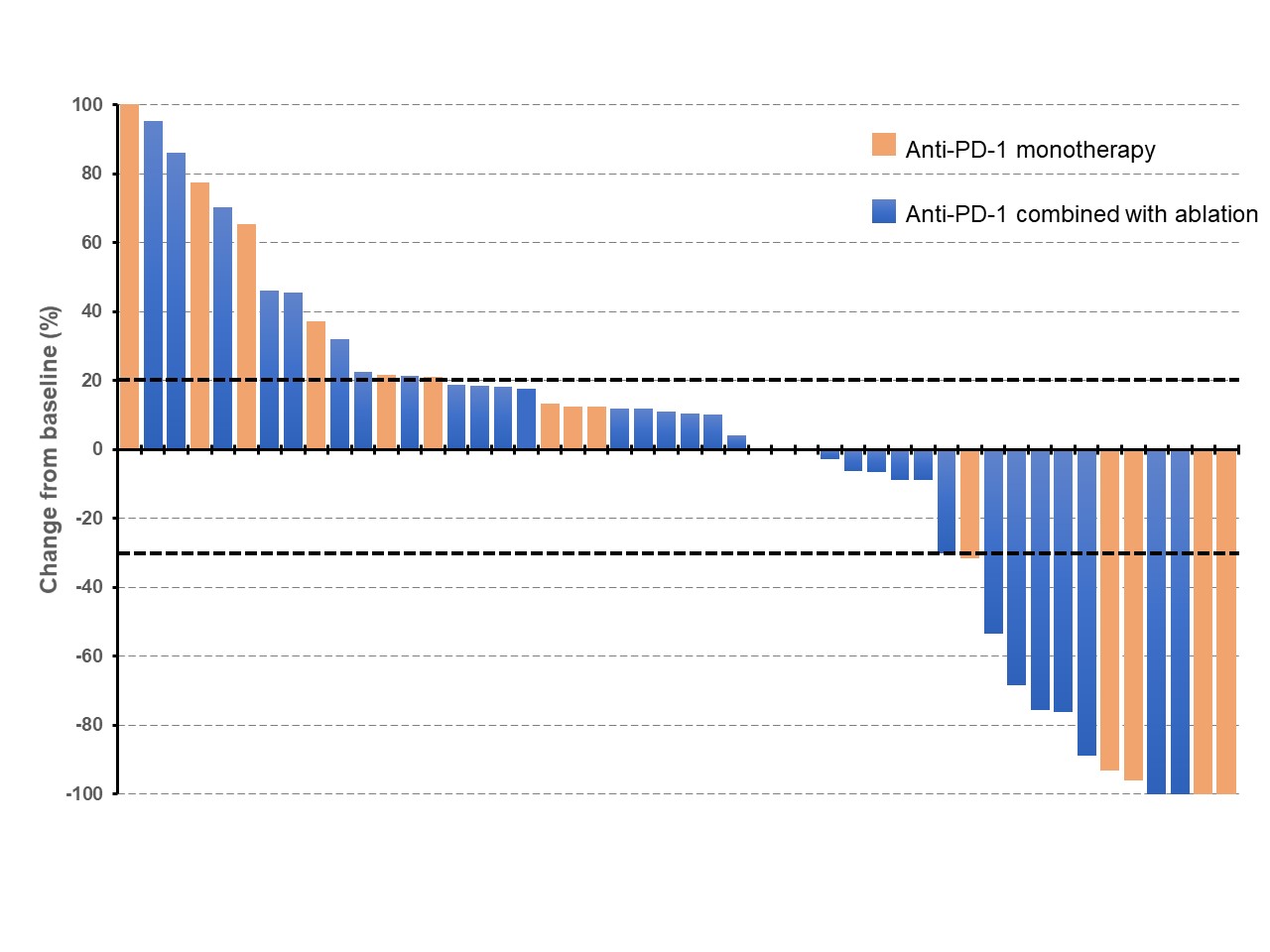


**Fig. S1.** **The waterfall plot shows the best percentage changes from baseline in the size of target tumors.** Assessed in the 48 patients with image examinations at baseline and after treatment. PD-1, programmed cell death protein-1.


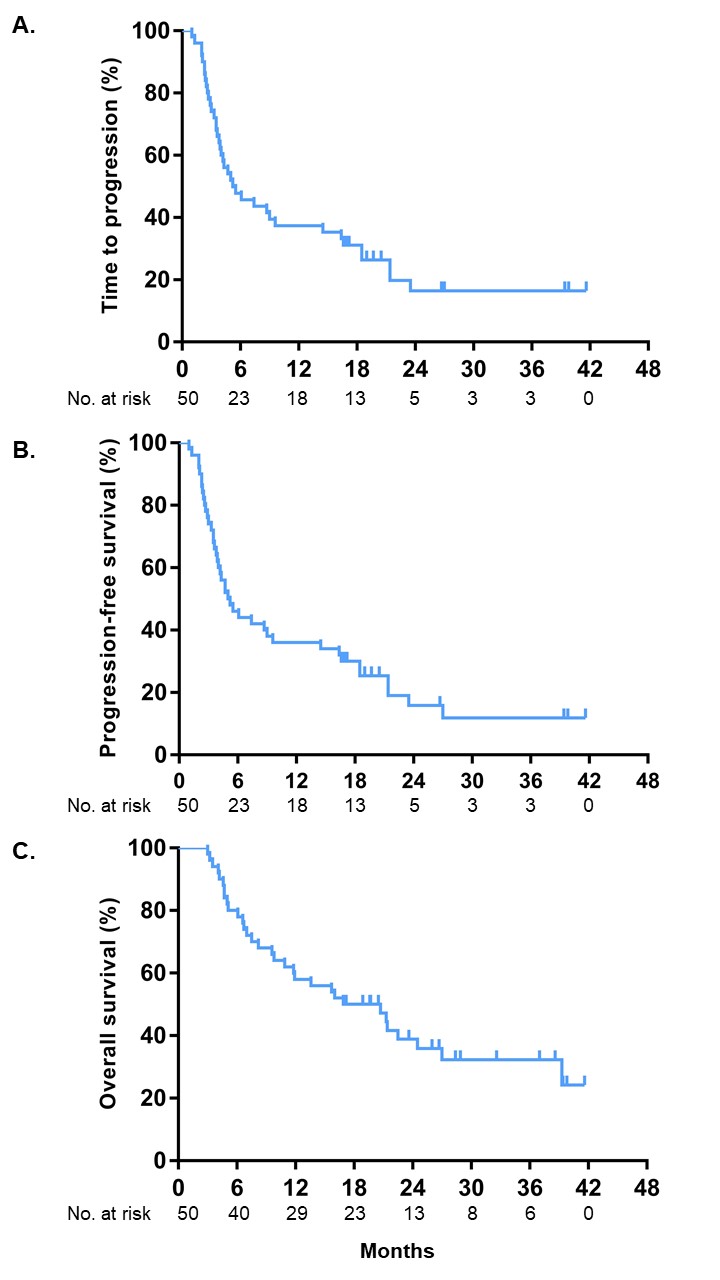


**Fig. S2. Kaplan-Meier estimates of survival curves. (A) Time to progression. (B) Progression-free survival. (C) Overall survival.**

**
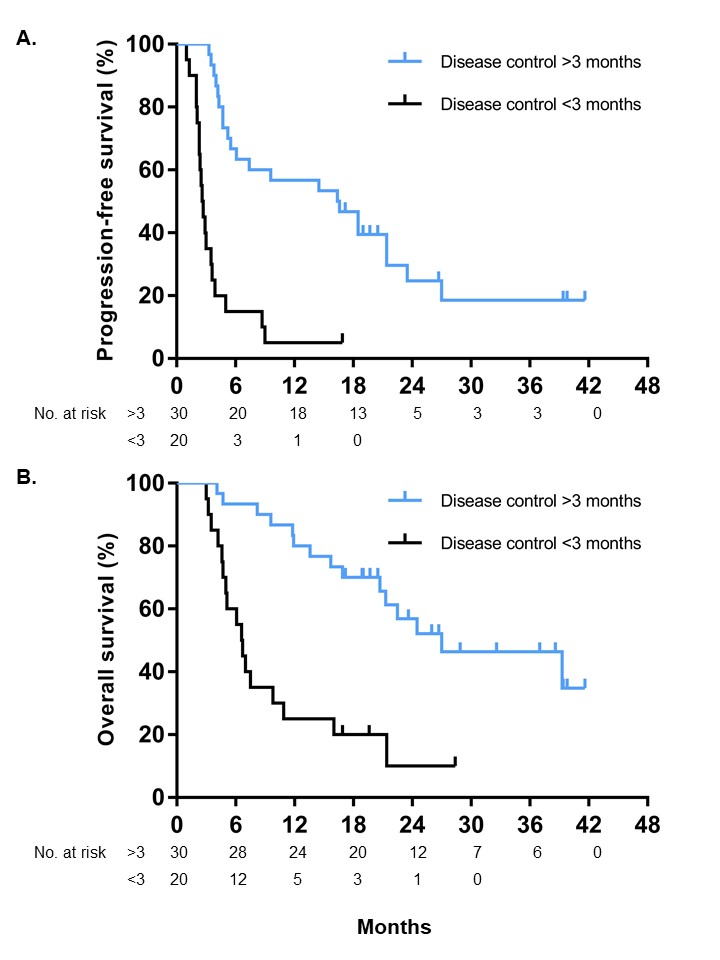
**

**Fig. S3. Kaplan-Meier curves show that both the Progression-free and overall survival in patients with disease control time more than three months was longer than those in patients who without (P <0.001 for both). (A) Progression-free survival. (B) Overall survival.**

**
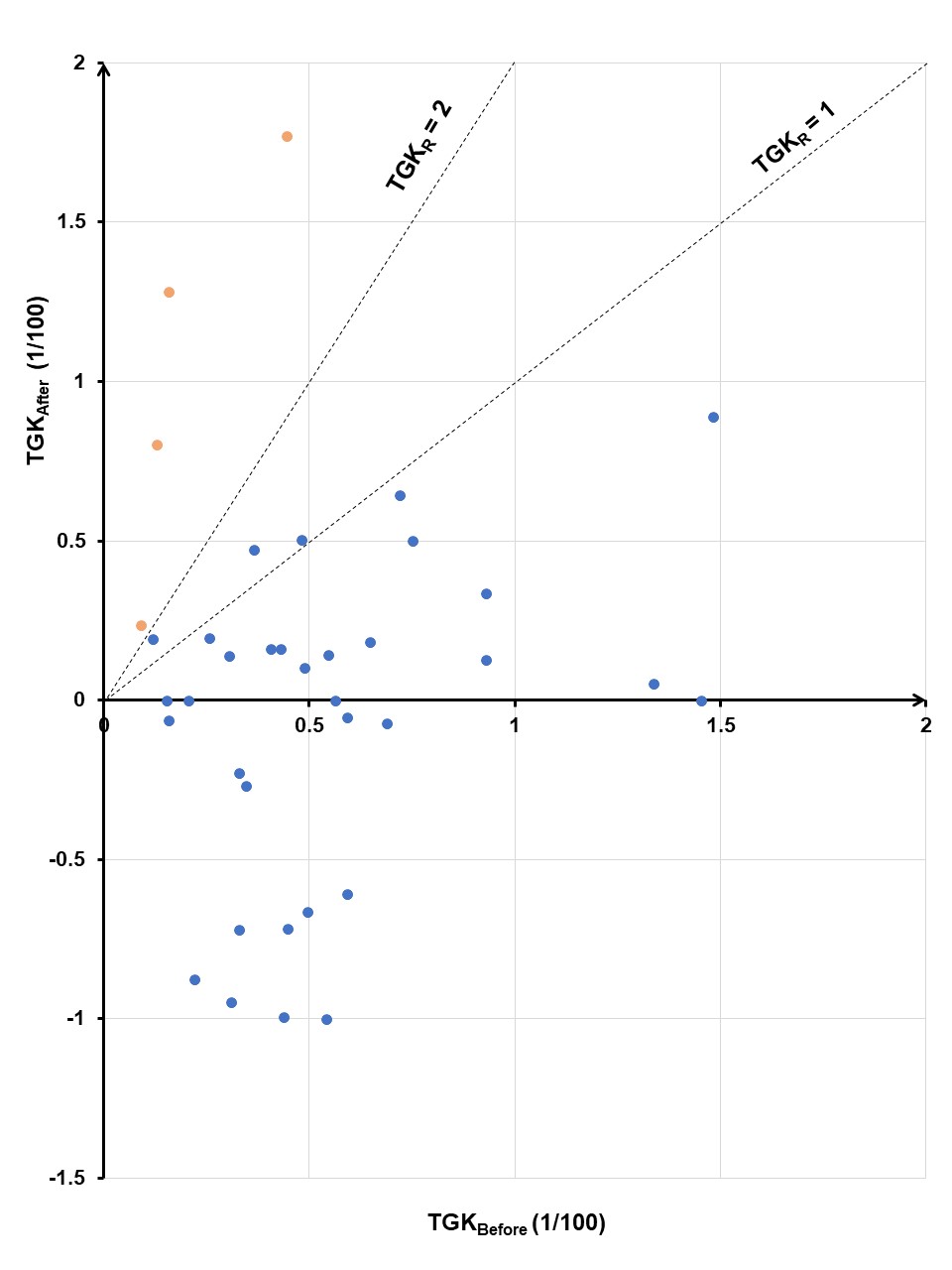
**

**Fig. S4.** **A scatter plot shows the tumor growth kinetics before (TGK_Before_) and after (TGK_After_) anti-PD-1 treatment.** Assessed in the 41 patients with eligible image examinations before anti-PD-1 treatment, at baseline, and after treatment. PD-1, programmed cell death protein-1; TGK, tumor growth kinetics; TGK_R_, TGK ratio.
